# Antibody evolution to SARS-CoV-2 after single-dose Ad26.COV2.S vaccine in humans

**DOI:** 10.1084/jem.20220732

**Published:** 2022-07-01

**Authors:** Alice Cho, Frauke Muecksch, Zijun Wang, Tarek Ben Tanfous, Justin DaSilva, Raphael Raspe, Brianna Johnson, Eva Bednarski, Victor Ramos, Dennis Schaefer-Babajew, Irina Shimeliovich, Juan P. Dizon, Kai-Hui Yao, Fabian Schmidt, Katrina G. Millard, Martina Turroja, Mila Jankovic, Thiago Y. Oliveira, Anna Gazumyan, Christian Gaebler, Marina Caskey, Theodora Hatziioannou, Paul D. Bieniasz, Michel C. Nussenzweig

**Affiliations:** 1 Laboratory of Molecular Immunology, The Rockefeller University, New York, NY; 2 Laboratory of Retrovirology, The Rockefeller University, New York, NY; 3 Howard Hughes Medical Institute, Chevy Chase, MD

## Abstract

The single-dose Ad.26.COV.2 (Janssen) vaccine elicits lower levels of neutralizing antibodies and shows more limited efficacy in protection against infection than either of the two available mRNA vaccines. In addition, Ad.26.COV.2 has been less effective in protection against severe disease during the Omicron surge. Here, we examined the memory B cell response to single-dose Ad.26.COV.2 vaccination. Compared with mRNA vaccines, Ad.26.COV.2 recipients had significantly lower numbers of RBD-specific memory B cells 1.5 or 6 mo after vaccination. Despite the lower numbers, the overall quality of the memory B cell responses appears to be similar, such that memory antibodies elicited by both vaccine types show comparable neutralizing potency against SARS-CoV-2 Wuhan-Hu-1, Delta, and Omicron BA.1 variants. The data help explain why boosting Ad.26.COV.2 vaccine recipients with mRNA vaccines is effective and why the Ad26.COV2.S vaccine can maintain some protective efficacy against severe disease during the Omicron surge.

## Teaser

Cho et al. describe the antibody immune response after a single dose of the Ad26.COV2.S vaccine compared with mRNA vaccines. They find that Ad26.COV2.S vaccination produces fewer memory B cells but with a potency and breadth comparable to those found after mRNA vaccination.

## Introduction

Severe acute respiratory syndrome coronavirus (SARS-CoV-2) produced a worldwide pandemic, infecting >470 million people and causing >6 million deaths. In the United States, the US Food and Drug Administration (FDA) authorized the use of three vaccines encoding prefusion-stabilized SARS-CoV-2 spike: two mRNA-based, BNT162b2 from Pfizer-BioNTech and mRNA-1273 from Moderna, and an adenovirus-based vaccine, Ad26.COV2.S from Janssen (Hsieh et al., 2020 *Preprint*). While both mRNA-based vaccines were initially approved as two-dose primary vaccine regimens, the replication-incompetent adenovirus 26 (Ad26) vector–based Ad26.COV2.S vaccine received FDA emergency authorization as a single-dose vaccine.

Despite the clear benefits of vaccination, the FDA recommends limited use of the Ad26.COV2.S vaccine due to emerging concerns about the risk of vaccine-associated thrombocytopenia syndrome (Food and Drug Administration, 2022). All three vaccines have proven effective, with substantial protection against COVID-19 infection, hospitalization, and death (Botton et al., 2022; Self et al., 2021). However, protection against COVID-19 infection appeared to wane over time with Ad26.COV2.S, which showed a decrease from 75 to 60% protective efficacy 5 mo after vaccination, compared with a decrease in vaccine efficacy from 95% to either 67 or 80% after BNT162b2 and mRNA-1273 vaccination, respectively, over a similar period of time (Lin et al., 2022). Loss of protection against infection was associated with lower overall levels of SARS-CoV-2 spike (S) protein–specific antibodies and plasma neutralizing activity after Ad26.COV2.S immunization compared with mRNA vaccines for ≤6 mo after vaccination (Collier et al., 2021; Sadoff et al., 2022).

In contrast to protection from infection, Wuhan-Hu-1–based mRNA vaccines maintain effectiveness against hospitalization and death even in the face of infection with SARS-CoV-2 antigenic variants (Andrews et al., 2022; Lin et al., 2022; Zheutlin et al., 2022 *Preprint*). While some protective efficacy against hospitalization and death caused by variants of concern can be observed for up to 5 mo after Ad.26.COV2 immunization, efficacy appears lower than for mRNA vaccines. The data are consistent with the finding that neutralizing titers elicited by single-dose Ad.26.COV2 immunization are lower compared with other vaccines (Bekker et al., 2022; GeurtsvanKessel et al., 2022; Rosenberg et al., 2022). Protection from severe disease by mRNA vaccines is attributed in part to a diverse collection of memory B cells that develop cross reactivity against viral variants over time (Muecksch et al., 2022). Far less is known about the evolution of the memory B cell response after Ad.26.COV2 vaccination or how they might contribute to protection over time. Here, we report on memory B cell evolution over a 6-mo period in a cohort of SARS-CoV-2–naive individuals after Ad.26.COV2 immunization.

## Results

We studied the immune response to a single dose of the Ad26.COV2.S (Janssen) vaccine in a cohort of 18 volunteers with no prior history of SARS-CoV-2 infection, recruited between April 26, 2021 and August 16, 2021, for sequential blood donations 1.5 mo (median 46 d, range 27–72 d) and 6 mo (median 179 d, range 136–200 d) after vaccination. Volunteers ranged in age from 23 to 56 yr and were 56% female and 44% male (for details, see Materials and methods and Table S1). Demographic information for the mRNA vaccinees (Cho et al., 2021; Muecksch et al., 2022; Wang et al., 2021c) and convalescent individuals (Gaebler et al., 2021; Robbiani et al., 2020) can be found in Table S2 (see Materials and methods).

### Plasma binding and neutralization

**Figure 1.**
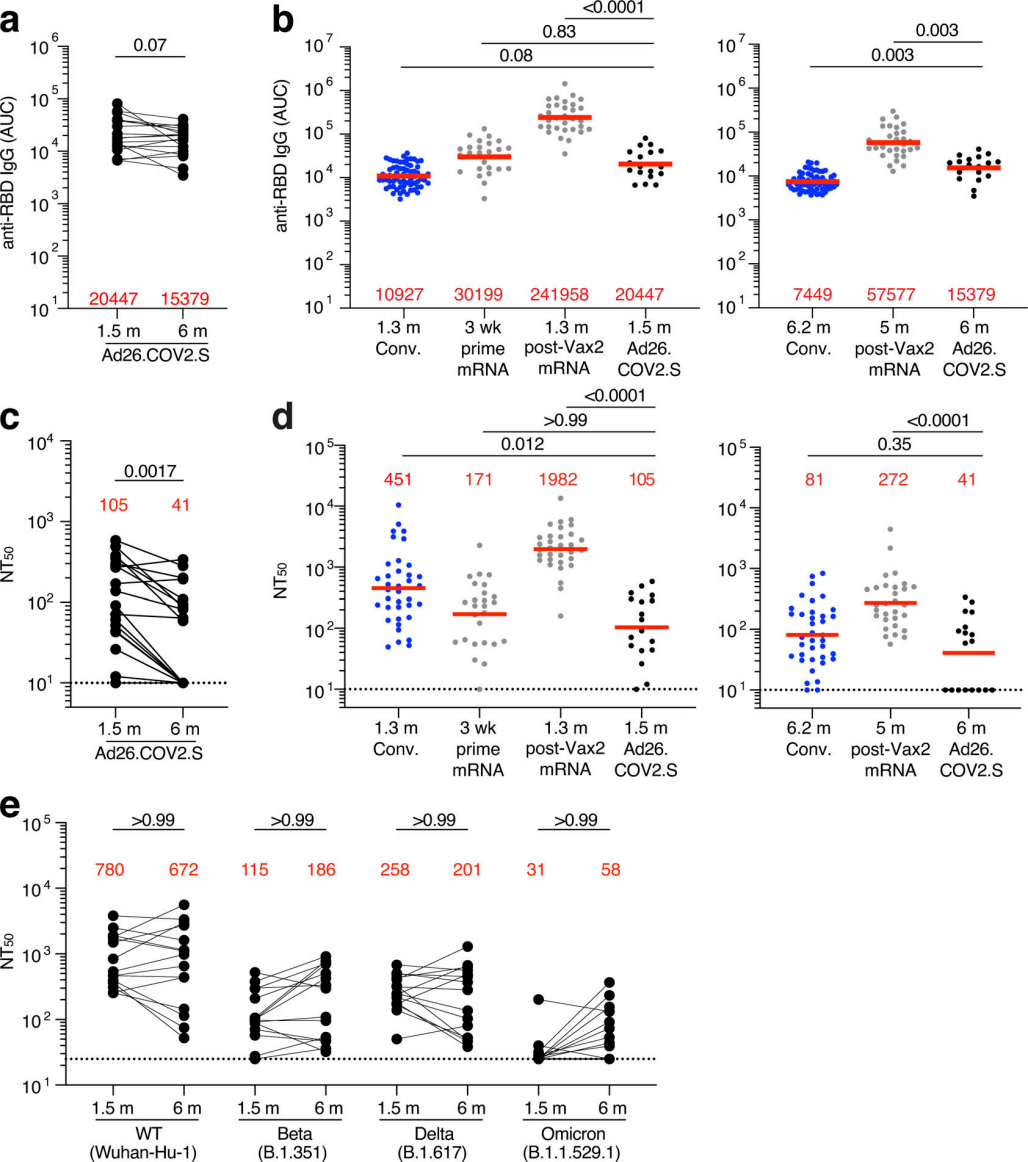
**Plasma ELISAs and neutralizing activity. (a)** Graph shows area under the curve (AUC) for plasma IgG antibody binding to SARS-CoV-2 Wuhan-Hu-1 RBD 1.5 mo (m) and 6 mo after vaccination for *n* = 18 samples. Lines connect longitudinal samples. **(b)** Graph shows AUC for plasma IgG binding to RBD in convalescent infected individuals 1.3 mo after infection (blue; Robbiani et al., 2020) and mRNA vaccinees (gray) after prime (3 wk after first vaccination) or 1.3 mo after second vaccination (Vax2; Cho et al., 2021) compared with Ad26.COV2.S vaccinees 1.5 mo after vaccination (left) or convalescent infected individuals 6.2 mo after infection (Gaebler et al., 2021) and mRNA vaccinees 5 mo after Vax2 (Cho et al., 2021) compared with Ad26.COV2.S vaccinees at 6 mo after vaccination (right). **(c)** Graph shows anti–SARS-CoV-2 NT_50_ values of plasma measured by a SARS-CoV-2 pseudotype virus neutralization assay in 293TAce2 cells (Robbiani et al., 2020; Schmidt et al., 2020) using WT (Wuhan Hu-1; Wu et al., 2020) SARS-CoV-2 pseudovirus (Robbiani et al., 2020; Schmidt et al., 2020) in plasma samples shown in panel a. **(d)** NT_50_ values of plasma measured by pseudotype virus neutralization assay comparing Ad26.COV2.S vaccinees to convalescent infected individuals (Gaebler et al., 2021; Robbiani et al., 2020) and mRNA vaccinees (Cho et al., 2021) either 1.5 mo (left) or 6 mo (right) after exposure, similar to plasma samples show in panel b. **(e)** Plasma neutralizing activity against indicated SARS-CoV-2 variants of interest/concern for *n* = 15 randomly selected samples assayed in HT1080Ace2 cl.14 cells. Wuhan-Hu-1 and Omicron BA.1 NT_50_ values are derived from Schmidt et al. (2022). See Materials and methods for a list of all substitutions/deletions/insertions in the spike variants. Deletions/substitutions corresponding to viral variants were incorporated into a spike protein that also includes the R683G substitution, which disrupts the furin cleavage site and increases particle infectivity. A corresponding WT control containing the R683G substitution was used in panel e. All experiments were performed at least in duplicate and repeated twice. Red bars and values represent geometric mean values. Statistical significance was determined by Wilcoxon matched-pairs signed rank test (a and c), two-tailed Kruskal–Wallis test with subsequent Dunn’s multiple comparisons (b and d), or Friedman test with subsequent Dunn’s multiple comparisons (e).

**Figure S1.**
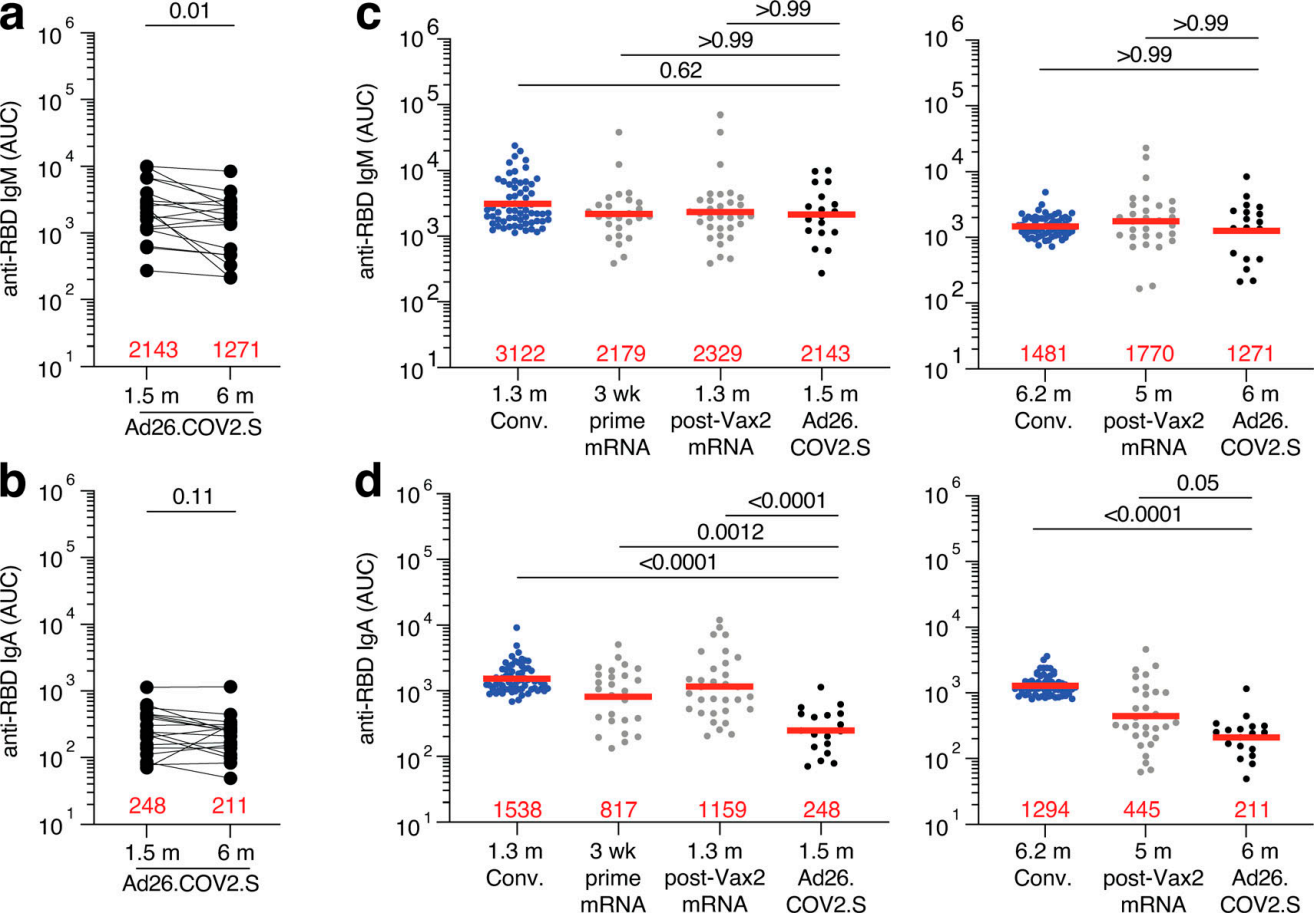
**Plasma ELISA. (a and b)** Graph shows area under the curve (AUC) for plasma IgM (a) and plasma IgA (b) antibody binding to SARS-CoV-2 Wuhan-Hu-1 RBD 1.5 mo (m) and 6 mo after vaccination for *n* = 18 samples. Lines connect longitudinal samples. **(c and d)** Graph shows AUC for plasma IgM (c) and plasma IgA (d) binding to RBD in convalescent infected individuals 1.3 mo after infection (Robbiani et al., 2020), and mRNA vaccinees after prime or 1.3 mo after second vaccination (Vax2; Cho et al., 2021) compared with Ad26.COV2.S vaccinees 1.5 mo after vaccination (left) or convalescent infected individuals 6.2 mo after infection (Gaebler et al., 2021) and mRNA vaccinees 5 mo after Vax2 (Cho et al., 2021) compared with Ad26.COV2.S vaccinees 6 mo after vaccination (right). All experiments were performed at least in duplicate and repeated twice. Red bars and values represent geometric mean values. Statistical significance in panels a and b was determined by Wilcoxon matched-pairs signed rank test, and in panels c and d was determined by two-tailed Kruskal–Wallis test with subsequent Dunn’s multiple comparisons.

Plasma antibody binding titers to SARS-CoV-2 receptor-binding domain (RBD) were measured by ELISA (Cho et al., 2021; Wang et al., 2021b). There was only a modest 1.3-fold decrease in geometric mean IgG-binding titers against RBD between 1.5 and 6 mo (P = 0.07, Fig. 1 a), compared with the significant 4.3-fold decrease reported for mRNA vaccinees at similar time points (Cho et al., 2021). RBD-binding IgG titers at the 1.5-mo time point were comparable to a single dose of the mRNA vaccine (collected after 3 wk) and to convalescents 1.3 mo after symptom onset (Cho et al., 2021; Robbiani et al., 2020; Fig. 1 b). After 6 mo, Ad26.COV2.S vaccine titers were significantly lower than in individuals who received two doses of an mRNA vaccine (Cho et al., 2021; P = 0.003; Fig. 1 b) but higher than convalescent infected individuals at a similar time after infection (Gaebler et al., 2021; P = 0.003; Fig. 1 b). IgM responses were comparable to both convalescent individuals and mRNA vaccinees, whereas IgA responses were significantly lower at both 1.5- and 6-mo time points compared with mRNA vaccinees and convalescent individuals (Fig. S1, a–d).

Neutralizing activity was determined for the same participants, using HIV-1 pseudotyped with Wuhan-Hu-1 SARS-CoV-2 S-protein (Cho et al., 2021; Wang et al., 2021b; Table S1). 1.5 mo after vaccination, individuals who received the Ad26.COV2.S vaccine had significantly lower neutralizing titers than either convalescents or vaccinees who received two doses of an mRNA vaccine (P = 0.012 and P < 0.0001, respectively; Fig. 1 d). In contrast to reports that neutralizing titers increase marginally over time in Ad26.COV2.S vaccinees, there was a modest but significant 2.7-fold decrease in geometric mean neutralizing titers after 6 mo in this cohort of 18 individuals (Barouch et al., 2021; GeurtsvanKessel et al., 2022; Sablerolles et al., 2022; Zhang et al., 2022 *Preprint*; P = 0.0017; Fig. 1 c). As a result, 39% of the participants receiving the Ad26.COV2.S vaccine had half-maximal neutralizing titers (NT_50_) that were below the limit of detection in our assay (NT_50_ < 10) 6 mo after vaccination. At that time point, the neutralizing activity was comparable to convalescents but remained significantly lower than individuals who had received two doses of an mRNA vaccine (P < 0.0001; Fig. 1 d).

Plasma neutralizing activity for 15 randomly selected samples was also assessed against SARS-CoV-2 variants using pseudotype viruses with variant spikes (Cho et al., 2021; Wang et al., 2021c; Table S3). Consistent with other reports (Barouch et al., 2021; Liu et al., 2022), at 1.5 mo neutralizing titers against Beta, Delta, and Omicron BA.1 were 6.8-, 3-, and 25-fold lower than Wuhan-Hu-1, respectively, and did not change significantly after 6 mo (Fig. 1 e).

### Memory B cell responses to SARS-CoV-2 RBD and N-terminal domain (NTD)

**Figure 2.**
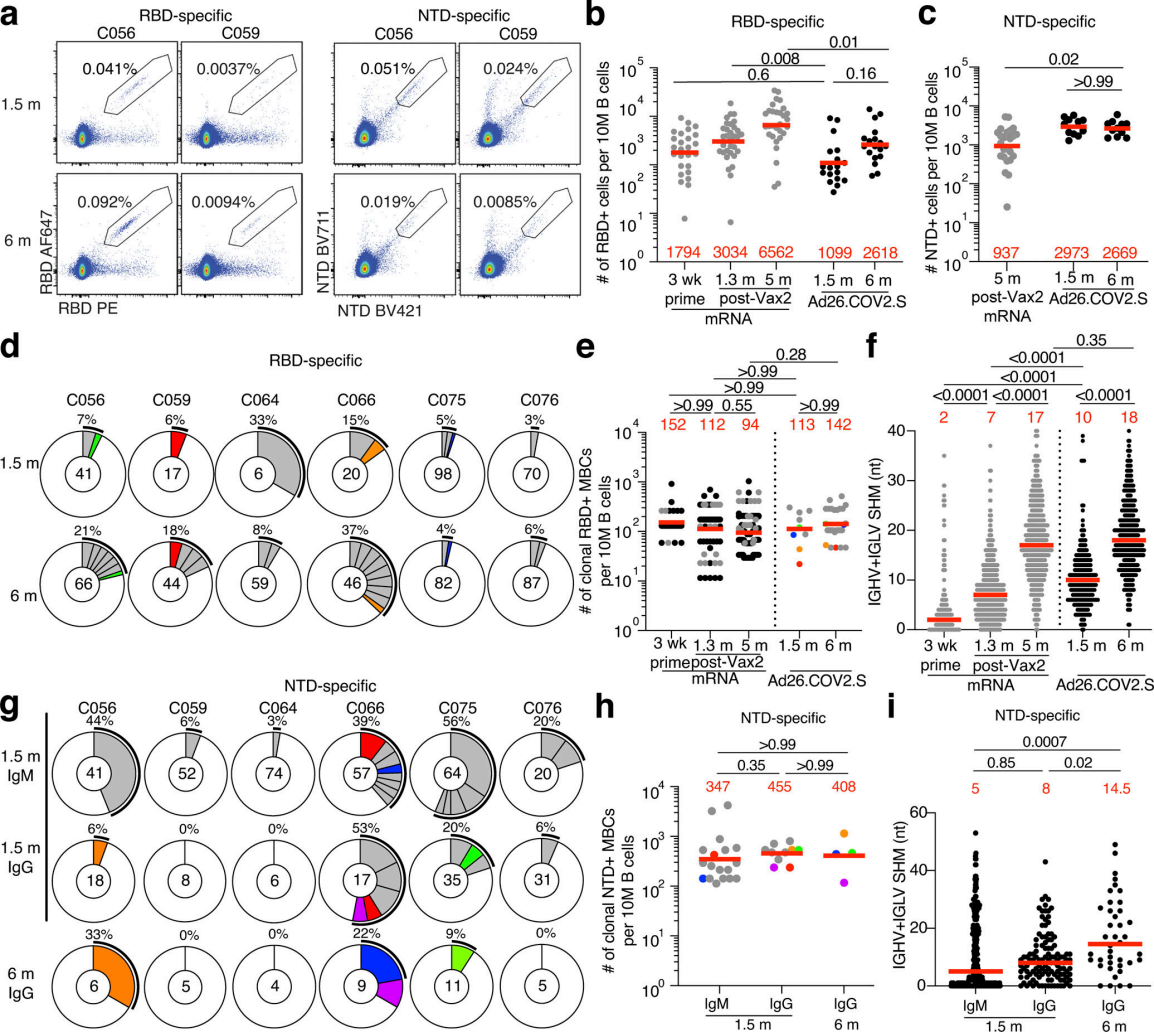
**Anti–SARS-CoV-2 RBD and NTD B cells after vaccination. (a)** Representative flow cytometry plots showing dual Alexa Fluor 647– and PE-Wuhan-Hu-1 RBD-binding (left) and BrilliantViolet-711- and BrilliantViolet-421-Wuhan-Hu-1 NTD-binding (right), single-sorted B cells from two individuals 1.5 mo (m) or 6 mo after vaccination. Gating strategy shown in Fig. S2. Percentage of antigen-specific B cells is indicated. **(b)** Graph summarizing the number of Wuhan-Hu-1 RBD-specific B cells per 10 million (M) B cells in Ad26.COV2.S vaccinees 1.5 and 6 mo after vaccination (black dots, *n* = 18) compared with mRNA vaccinees at prime and 1.3 and 5 mo after Vax2 (Cho et al., 2021; gray dots). **(c)** Graph summarizing the number of Wuhan-Hu-1 NTD-specific B cells per 10 million B cells in Ad26.COV2.S vaccinees 1.5 and 6 mo after vaccination (*n* = 18), compared with mRNA vaccinees 5 mo after Vax2 (gray dots). **(d)** Pie charts show the distribution of IgG antibody sequences obtained from Wuhan-Hu-1 RBD-specific memory B cells from six individuals 1.5 and 6 mo after vaccination. Time points indicated to the left of the charts. The number inside the circle indicates the number of sequences analyzed for the individual denoted above the circle. Pie slice size is proportional to the number of clonally related sequences. The black outline and associated numbers indicate the percentage of clonally expanded sequences detected at each time point. Colored slices indicate persisting clones (same *IGHV* and *IGLV* genes with highly similar CDR3s; see Materials and methods) found at more than one time point within the same individual. Gray slices indicate expanded clones unique to the time point. White slice represents sequences isolated only once. **(e)** Graph shows the number of clonally expanded RBD-specific MBCs per 10 million B cells. Left panel represent clones from mRNA vaccinees after prime or 1.3 and 5 mo after Vax2 (black dots represent persisting clones; gray dots represent unique clones; Muecksch et al., 2022). Right panel show clones from Ad26.COV2.S vaccinees 1.5 or 6 mo after vaccination, with each dot representing one clone illustrated in panel d (color dots represent matched persisting clones; gray dots represent unique clones). **(f)** Number of nucleotide SHMs in *IGHV* and *IGLV* in RBD-specific sequences 1.5 or 6 mo after vaccination, compared with mRNA vaccinees (gray) after prime, or 1.3 and 5 mo after Vax2 (Cho et al., 2021). **(g)** Pie charts showing distribution of IgM and IgG Wuhan-Hu-1 NTD-specific sequences 1.5 and 6 mo after vaccination from the same individuals as shown in panel d. Isotype and time point is indicated to left of graphs. **(h)** Graph shows the number of clonally expanded NTD-specific MBCs per 10 million B cells, with each dot representing one clone illustrated in panel g (color dots represent matched persisting clones; gray dots represent unique clones). **(i)** Number of nucleotide SHMs in *IGHV* and *IGLV* in NTD-specific sequences 1.5 or 6 mo after vaccination. Red bars and numbers represent geometric mean (b, c, e, and h) or median (f and i) values. Statistical difference was determined by two-tailed Kruskal–Wallis test with subsequent Dunn’s multiple comparisons (b, c, e, f, h, and i).

**Figure S2.**
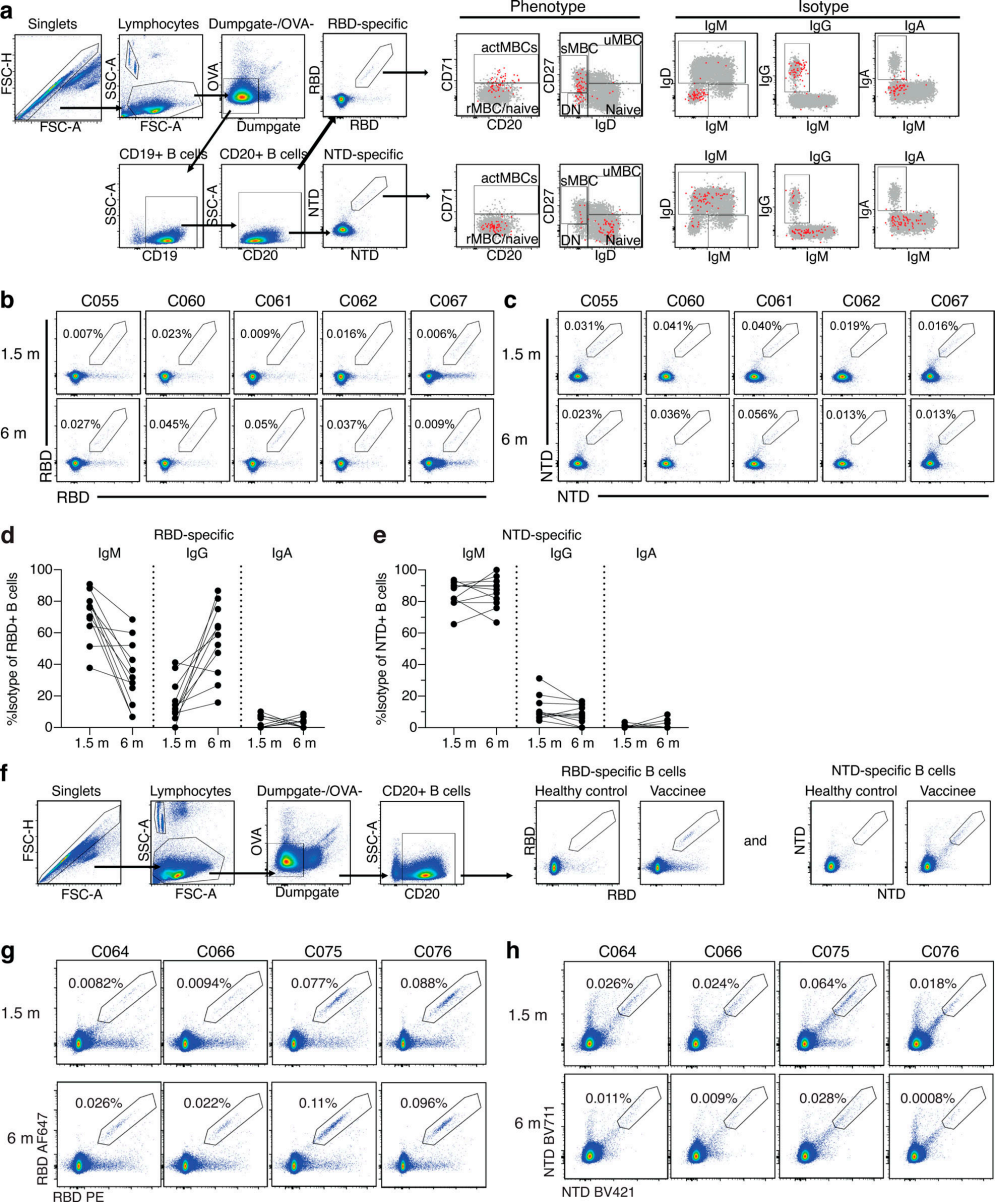
**Flow cytometry. (a)** Gating strategy for phenotyping. Gating was on lymphocytes singlets that were CD19^+^ or CD20^+^ and CD3^−^CD8^−^CD16^−^Ova^−^. Anti-IgG, IgM, IgA, IgD, CD71, and CD27 antibodies were used for B cell phenotype analysis. Antigen-specific cells were detected based on binding to Wuhan-Hu-1 RBD-PE^+^ and RBD-AF647^+^ or to Wuhan-Hu-1 NTD-BV711^+^ and NTD-BV421^+^. Counting beads were added to each sample and gated based on forward scatter (FSC) and side scatter (SSC) as per manufacturer instructions. **(b and c)** Representative flow cytometry plots of RBD-binding B cells (b) or NTD-binding B cells (c) in five individuals 1.5 and 6 mo after vaccination. **(d and e)** Graph showing the frequency of IgM, IgG, and IgA isotype in RBD-specific B cells (d) and NTD-specific B cells (e) 1.5 or 6 mo after vaccination. **(f)** Gating strategy for single-cell sorting for CD20^+^ B cells for Wuhan-Hu-1 RBD-PE and RBD-AF647 or Wuhan-Hu-1 NTD-BV711 and NTD-BV421. **(g and h)** Representative flow cytometry plots showing dual Alexa Fluor 647– and PE-Wuhan-Hu-1 RBD binding (g) and BrilliantViolet-711- and BrilliantViolet-421-Wuhan-Hu-1 NTD binding (h); single-cell-sorted B cells from four additional individuals 1.5 or 6 mo after vaccination. Percentage of antigen-specific B cells is indicated.

Memory B cells contribute to long-term immune protection from serious disease by mediating rapid, anamnestic recall antibody responses (Inoue et al., 2021). To examine the development of memory after Ad26.COV2.S vaccination, we initially enumerated B cells expressing surface receptors binding to the RBD or NTD of the SARS-CoV-2 spike protein using fluorescently labeled proteins (Fig. 2 a and Fig. S2, a–c). The number of RBD-binding memory B cells 1.5 mo after Ad26.COV2.S vaccination was significantly lower than for mRNA vaccinees 1.3 mo after the second mRNA vaccine dose (P = 0.008; Fig. 2 b; Cho et al., 2021). Although the number of RBD-binding memory cells increased 1.5–6 mo after the single-dose Ad26.COV2.S vaccine, the number remained lower than after mRNA vaccination at a similar time point (P = 0.01; Fig. 2 b). NTD-specific memory B cells had been found to persist in mRNA vaccinees, slightly increasing at 6 mo after vaccination (Goel et al., 2021, 2022). In contrast, the number of NTD-binding memory B cells did not change between the two time points after Ad26.COV2.S vaccination and was significantly higher than after mRNA 5–6 mo after vaccination (P = 0.02; Fig. 2 c). Additional phenotyping showed that RBD-specific memory B cells elicited by the Ad26.COV2.S vaccine showed the expected switch from IgM to IgG (Fig. S2 d).

**Figure S3.**
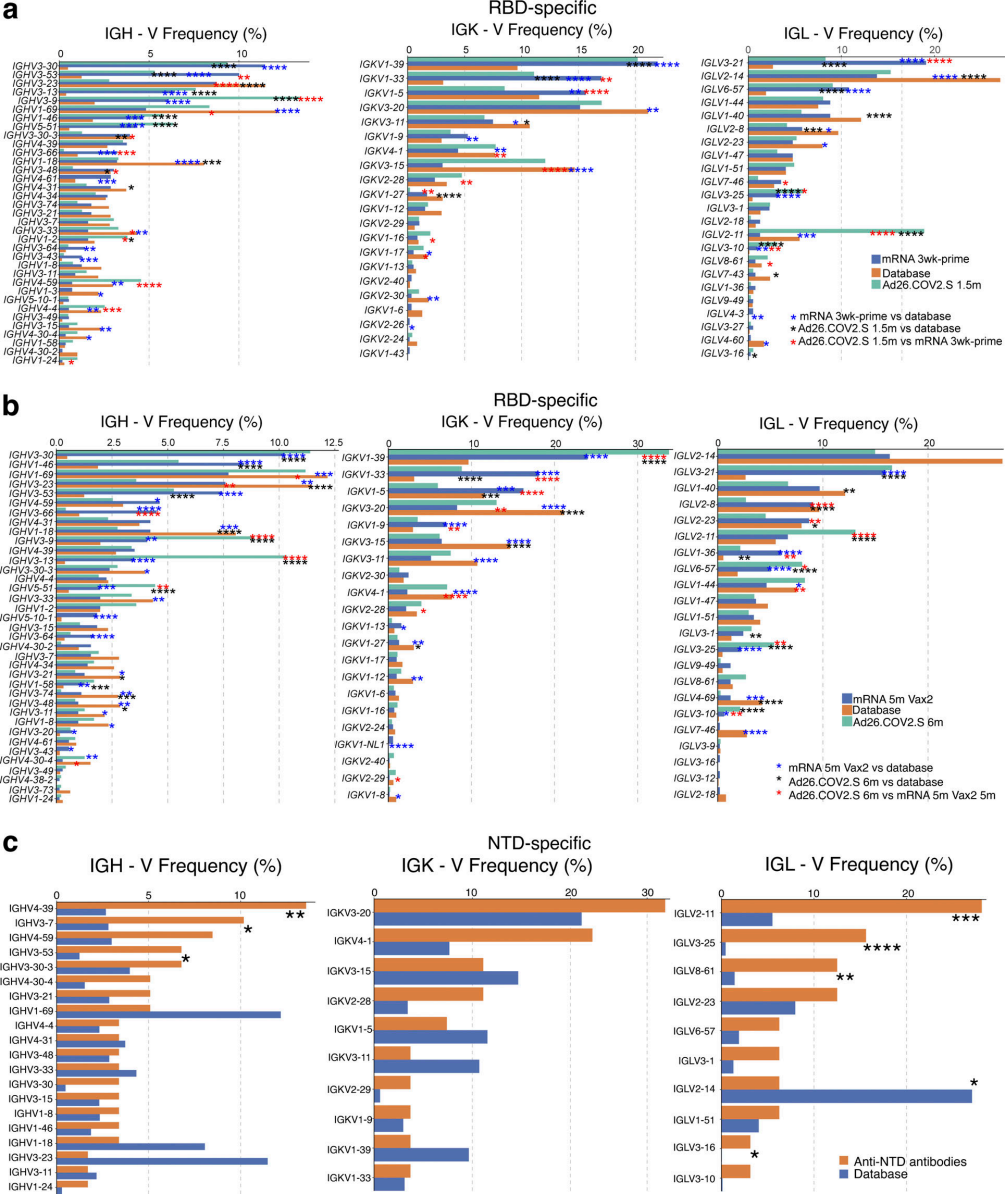
**Frequency distribution of human V genes in SARS-CoV-2 RBD- and NTD-binding B cells. (a and b)** Comparison of the frequency distribution of human V genes for heavy chain and light chains of anti-RBD antibodies from this study and from a database of shared clonotypes of human B cell receptor generated by Soto et al. (2019). Graph shows relative abundance of human *IGHV* (left), *IGKV* (middle), and *IGLV* (right) genes in Sequence Read Archive accession SRP010970 (orange), Ad26.COV2.S antibodies (green), and mRNA vaccinees (blue), comparing 1.5 mo after Ad26.COV2.S vaccination to 1.3 mo after one dose of mRNA vaccine (prime; a) or 6 mo after Ad26.COV2.S vaccination to 5 mo after second dose of mRNA vaccine (b). Statistical significance was determined by two-sided binomial test. *, P ≤ 0.05; **, P ≤ 0.01; ***, P ≤ 0.001; ****, P ≤ 0.0001. Color of stars: black, Ad26.COV2.S vaccination vs. human database; blue, mRNA vaccination vs. human database; red, Ad26.COV2.S vaccination vs. mRNA vaccination. **(c)** Comparison of the frequency distribution of human V genes for heavy chain and light chains of all anti-NTD antibodies from this study to a database of shared clonotypes of human B cell receptor generated by Soto et al. (2019). Graph shows relative abundance of human *IGHV* (left), *IGKV* (middle), and *IGLV* (right panel) genes in Sequence Read Archive accession SRP010970 (blue) Ad26.COV2.S antibodies (orange). Statistical significance was determined by two-sided binomial test. *, P ≤ 0.05; **, P ≤ 0.01; ***, P ≤ 0.001; ****, P ≤ 0.0001.

To examine the specificity and neutralizing activity of the antibodies produced by memory cells, we purified single antigen–specific B cells by baiting with both Wuhan-Hu-1 RBD and NTD proteins in six randomly selected individuals. Antibody genes were sequenced and produced the recombinant antibodies in vitro. 636 paired anti-RBD antibody sequences were obtained from six vaccinees sampled at the two time points after Ad26.COV2.S vaccination (Fig. 2 d and Table S4). Clonally expanded RBD-specific B cells represented 6.3 and 13.5% of all memory cells 1.5 and 6 mo after vaccination, respectively, similar to mRNA vaccination (Fig. 2, d and e). In addition, the same set of VH and VL genes were overrepresented between Ad26.COV2.S and mRNA vaccinees, including VH3-30, VH3-53, VK1-39, and VL3-21 (Fig. S3), suggesting that similar germline genes are engaged in response to RBD, regardless of the type of vaccination. However, very few clones persisted over time in Ad26.COV2.S vaccinees (13% of all clonal expansions detected in Fig. 2 d). The majority of expanded clones were found uniquely at one of the two time points (unique clones, 78%), suggesting ongoing memory B cell turnover (Fig. 2 d). When comparing accumulation of somatic mutations, Ad26.COV2.S vaccinees had higher levels of mutations at the 1.5-mo time point compared with mRNA vaccinees after prime or 1.3 mo after the second vaccine dose (P < 0.0001; Fig. 2 f). Continued memory B cell evolution was also evident in the accumulation of somatic mutations 1.5–6 mo after Ad26.COV2.S vaccination (P < 0.0001; Fig. 2 f), ultimately resulting in comparable levels of mutations between Ad26.COV2.S and mRNA vaccinees. Thus, although the absolute number of RBD-specific memory B cells 6 mo after a single dose of Ad26.COV2.S was lower than after two doses of an mRNA vaccine, the two showed indistinguishable proportions of clonally expanded RBD-specific memory B cells that carry equivalent numbers of somatic mutations in their antibody genes.

To analyze the NTD-specific memory B cell repertoire, we sequenced 463 paired anti-NTD antibodies from the same six individuals (Fig. 2 g and Table S4). The geometric mean number of clonally expanded NTD-specific memory cells was 4-fold greater than RBD-specific memory B cells after 1.5 mo and remained 2.8-fold higher after 6 mo (Fig. 2, e and h). Similar to natural infection (Wang et al., 2022 *Preprint*), VH4-39 and VH3-7 genes were overrepresented in the NTD-specific memory B cell repertoire elicited by the Ad26.COV2.S vaccine (Fig. S3). Expanded clones accounted for an average of 28 and 17% of the IgM and IgG repertoire 1.5 mo after vaccination, respectively, and 13% of the IgG repertoire after 6 mo. Like the RBD-specific memory B cells, only a minority (25%) of all expanded NTD-specific memory clones persisted between the two time points (Fig. 2 g), and continued evolution was evident by accumulation of somatic mutations over time (P = 0.02; Fig. 2 i). In conclusion, the NTD-specific memory B cell compartment elicited by one dose of the Ad26.COV2.S vaccine is moderately larger in size and clonality to its anti-RBD counterpart.

### Neutralizing activity of mAbs

**Figure 3.**
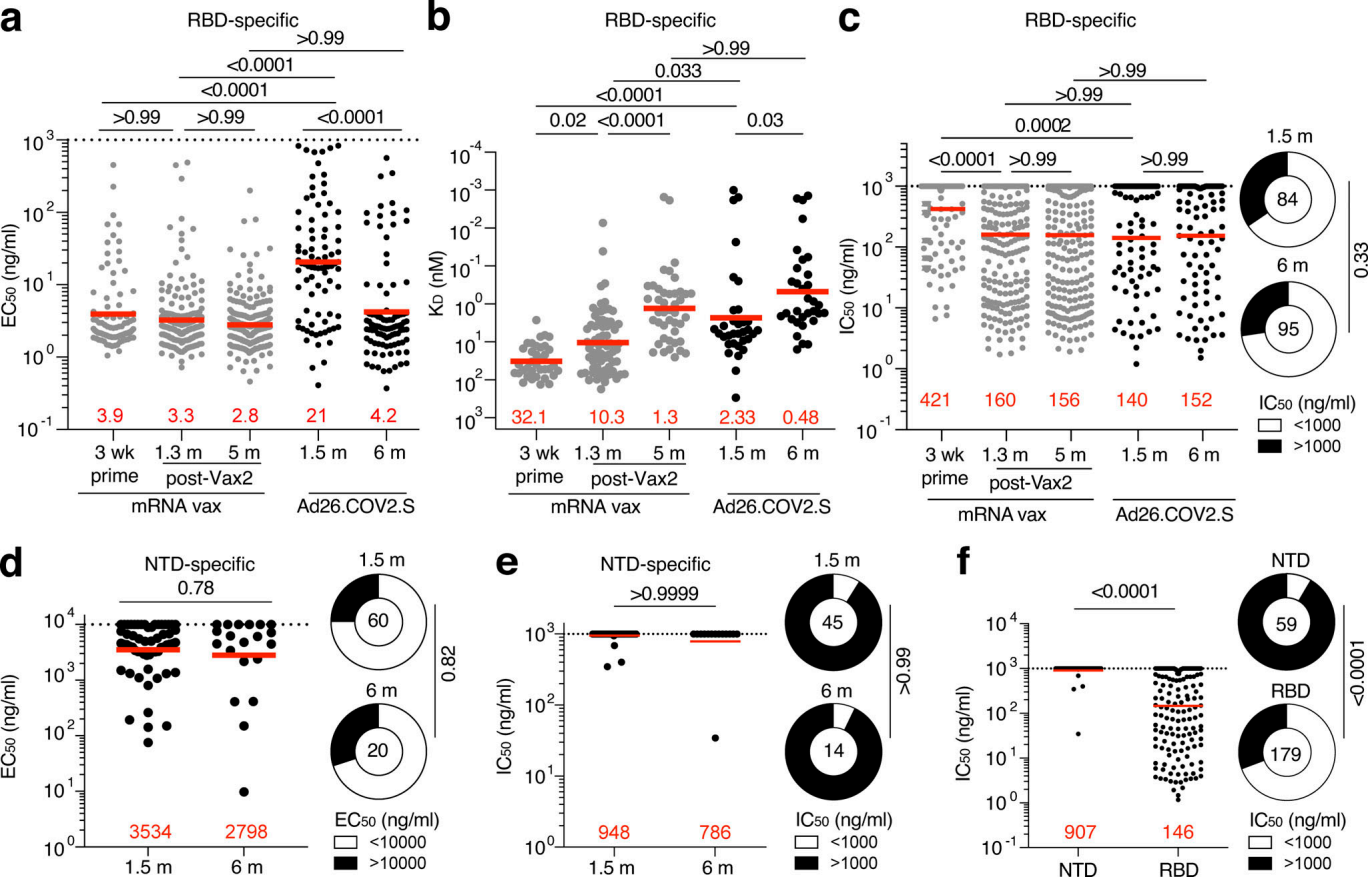
**Anti–SARS-CoV-2 mAbs. (a)** Graph shows EC_50_ of *n* = 179 Wuhan-Hu-1 RBD-binding mAbs measured by ELISA against Wuhan-Hu-1 RBD 1.5 and 6 mo after vaccination, compared with EC_50_ measured in mRNA vaccinees after prime or 1.3 and 5 mo after Vax2 (Cho et al., 2021; Muecksch et al., 2022). **(b)** Graph showing affinity measurements (*K*_*D*_) for Wuhan-Hu-1 RBD measured by BLI for antibodies cloned from mRNA vaccinees after prime and 1.3 and 6 mo after Vax2 (Cho et al., 2021; Muecksch et al., 2022) compared with antibodies cloned from Ad26.COV2.S vaccinees 1.5 and 6 mo after vaccination (*n* = 33, each). **(c)** Graphs show anti–SARS-CoV-2 neutralizing activity of mAbs measured by a SARS-CoV-2 pseudotype virus neutralization assay using WT (Wuhan Hu-1; Wu et al., 2020) SARS-CoV-2 pseudovirus (Robbiani et al., 2020; Schmidt et al., 2020) for antibodies cloned from mRNA vaccinees after prime and 1.3 and 5 mo after Vax2 (Cho et al., 2021; Muecksch et al., 2022) compared with antibodies cloned from Ad26.COV2.S vaccinees (*n* = 179) 1.5 and 6 mo after vaccination. Pie charts to the right indicate the frequency of neutralizing (IC_50_ < 1,000 ng/ml, white) vs. nonneutralizing antibodies (IC_50_ >1,000 ng/ml, black) cloned from Ad26.COV2.S vaccinees. **(d)** Graph showing EC_50_ of *n* = 80 mAbs measured by ELISA against Wuhan-Hu-1 NTD 1.5 and 6 mo after vaccination. Right: Pie charts indicating frequency of antibodies determined to bind (EC_50_ <10,000 ng/ml, white) or not bind (EC_50_ >10,000 ng/ml, black). **(e)** Graph showing IC_50_ of NTD-specific antibodies 1.5 and 6 m after vaccination. Right: Pie charts indicating frequency of SARS-CoV-2 WT pseudovirus neutralizing (IC_50_ <1,000 ng/ml, white) vs. nonneutralizing (IC_50_ >1,000 ng/ml, black) NTD-specific mAbs. **(f)** Graph comparing the IC_50_ of all NTD-specific mAbs (*n* = 80) and RBD-specific mAbs (*n* = 179) derived from Ad26.COV2.S vaccinees. Right: Pie charts indicating frequency of either NTD- or RBD-specific neutralizing (IC_50_ <1,000 ng/ml, white) vs. nonneutralizing (IC_50_ >1,000 ng/ml, black) mAbs. Red bars and lines indicate geometric mean values. All experiments were performed at least in duplicate and were repeated twice. Statistical significance was determined by two-tailed Kruskal–Wallis test with subsequent Dunn’s multiple comparisons (a–c) or by two-tailed Mann–Whitney *U* test (d–f). Pie charts were compared using two-tailed Fisher’s exact test.

192 anti-RBD mAbs were expressed and tested for binding by ELISA. 93% (*n* = 179) bound to the Wuhan-Hu-1 RBD, indicating the high efficiency of RBD-specific memory B cell isolation (Table S5). At the initial time point, the geometric mean ELISA half-maximal effective concentration (EC_50_) of the mAbs obtained from Ad26.COV2.S vaccinees was significantly higher than from individuals receiving a single dose of an mRNA vaccine (P = 0.0001; Fig. 3 a; Cho et al., 2021). However, the EC_50_ of RBD-binding antibodies elicited by the Ad26.COV2.S vaccine improved over time such that the antibodies elicited by the two vaccines had comparable EC_50_ values after 5–6 mo (Fig. 3 a).

Because EC_50_s are only an indirect measure of affinity, we performed biolayer interferometry (BLI) experiments on a subset of the antibodies (*n* = 33 each at 1.5 and 6 mo) to measure discrete dissociation constant (*K*_*D*_) values. Affinity was significantly higher among antibodies elicited by the Ad26.COV2.S vaccine compared with those obtained after the mRNA prime and second dose (P < 0.0001 and P = 0.03, respectively; Fig. 3 b; Cho et al., 2021). For both vaccine platforms, antibody affinity improved over time, reaching equivalent levels at the 5–6-mo time point (Fig. 3 b).

All 179 RBD-binding antibodies were tested for neutralization (84 and 95 antibodies isolated after 1.5 and 6 mo, respectively). Compared with the mRNA prime, memory antibodies elicited by the Ad26.COV2.S vaccine were significantly more potent against viruses pseudotyped with the Wuhan-Hu-1 RBD (half-maximal inhibitory concentration [IC_50_] 140 vs. 421 ng/ml; P = 0.0002; Fig. 3 c). However, the neutralizing activity of the anti-RBD memory antibodies elicited by mRNA vaccination improved after the second dose, and the two vaccines generated antibodies of equivalent potency after 5–6 mo (IC_50_ 152 vs. 156; P > 0.99; Fig. 3 c; Cho et al., 2021).

To examine the repertoire of NTD-specific memory B cells elicited by the Ad26.COV2.S vaccine, we expressed 60 and 20 antibodies obtained 1.5 and 6 mo after vaccination, respectively (Table S6). 59 bound to NTD with relatively poor EC_50_ values, with no significant difference between time points (Fig. 3 d and Table S6). When tested for neutralizing activity against Wuhan-Hu-1–pseudotyped virus (Wang et al., 2022 *Preprint*), only 4 of the 59 NTD-binding mAbs showed neutralizing activity, with no significant different between time points (Fig. 3 e). Thus, the overall frequency of memory B cells producing neutralizing anti-NTD antibodies is significantly lower than those producing anti-RBD (Fig. 3 f). We conclude that anti-NTD memory antibodies are likely to make a more modest contribution to neutralizing responses against SARS-CoV-2 than their anti-RBD counterparts.

### Epitope specificity of RBD-binding antibodies

**Figure 4.**
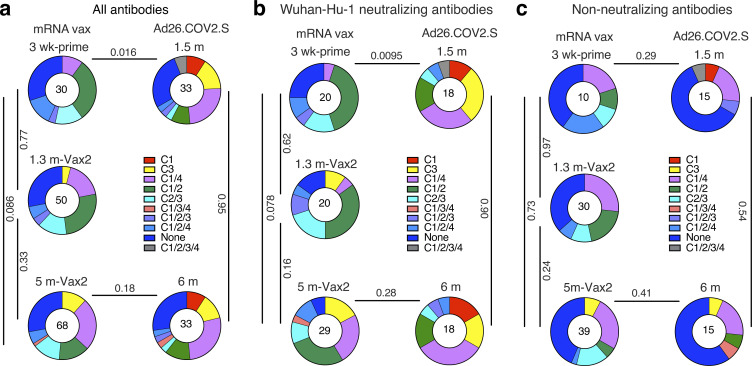
**Epitope mapping. (a–c)** Results of epitope mapping performed by competition BLI, comparing mAbs cloned from Ad26.COV2.S vaccinees 1.5 and 6 mo after vaccination (*n* = 33, each) to mAbs cloned from mRNA vaccinees at prime or 1.3 and 5 mo after Vax2 (Cho et al., 2021; Muecksch et al., 2022). Pie charts show the distribution of the antibody classes among all RBD-binding antibodies (a), Wuhan-Hu-1 neutralizing antibodies only (b), or nonneutralizing antibodies only (c). Statistical significance was determined by using a two-tailed χ^2^ test.

mRNA vaccination elicits anti-RBD antibodies that target four structurally defined classes of epitopes on the RBD (Barnes et al., 2020a; Muecksch et al., 2021, 2022; Wang et al., 2021b; Yuan et al., 2020). The relative distribution of epitopes targeted by RBD-binding antibodies can contribute to their potency and breadth. Whereas class 1 and 2 antibodies, that block ACE2 binding directly, tend to be more potent, class 3 and 4 target more conserved regions and can be broader (Gaebler et al., 2021; Muecksch et al., 2021, 2022; Wang et al., 2021b). To define the epitopes recognized by anti-RBD memory antibodies elicited by the Ad26.COV2.S vaccine, we performed BLI competition experiments. A preformed antibody-RBD complex was exposed to a second antibody targeting one of four classes of structurally defined epitopes (Barnes et al., 2020a; Robbiani et al., 2020; C105 as class 1; C144 as class 2; C135 as class 3; and C118 as class 1/4). We examined 33 random RBD-binding antibodies obtained from the 1.5- and 6-mo time points each, including 18 of 33 with IC_50_ values <1,000 ng/ml (Table S7). In contrast to the antibodies elicited after a single dose of an mRNA vaccine that primarily target class 1 and 2 epitopes, class 3 and 1/4 specific antibodies dominated the repertoire 1.5 mo after Ad26.COV2.S vaccination (P = 0.016, Fig. 4 a). This difference is particularly striking when considering neutralizing as opposed to nonneutralizing antibodies (Fig. 4, b and c). However, shifts in the repertoire of the mRNA vaccinees over time alleviated these differences, resulting in comparable epitope specificities in the two groups 5–6 mo after vaccination (Fig. 4, a and b; Cho et al., 2021; Muecksch et al., 2022).

### Neutralizing breadth of memory B cells

**Figure 5.**
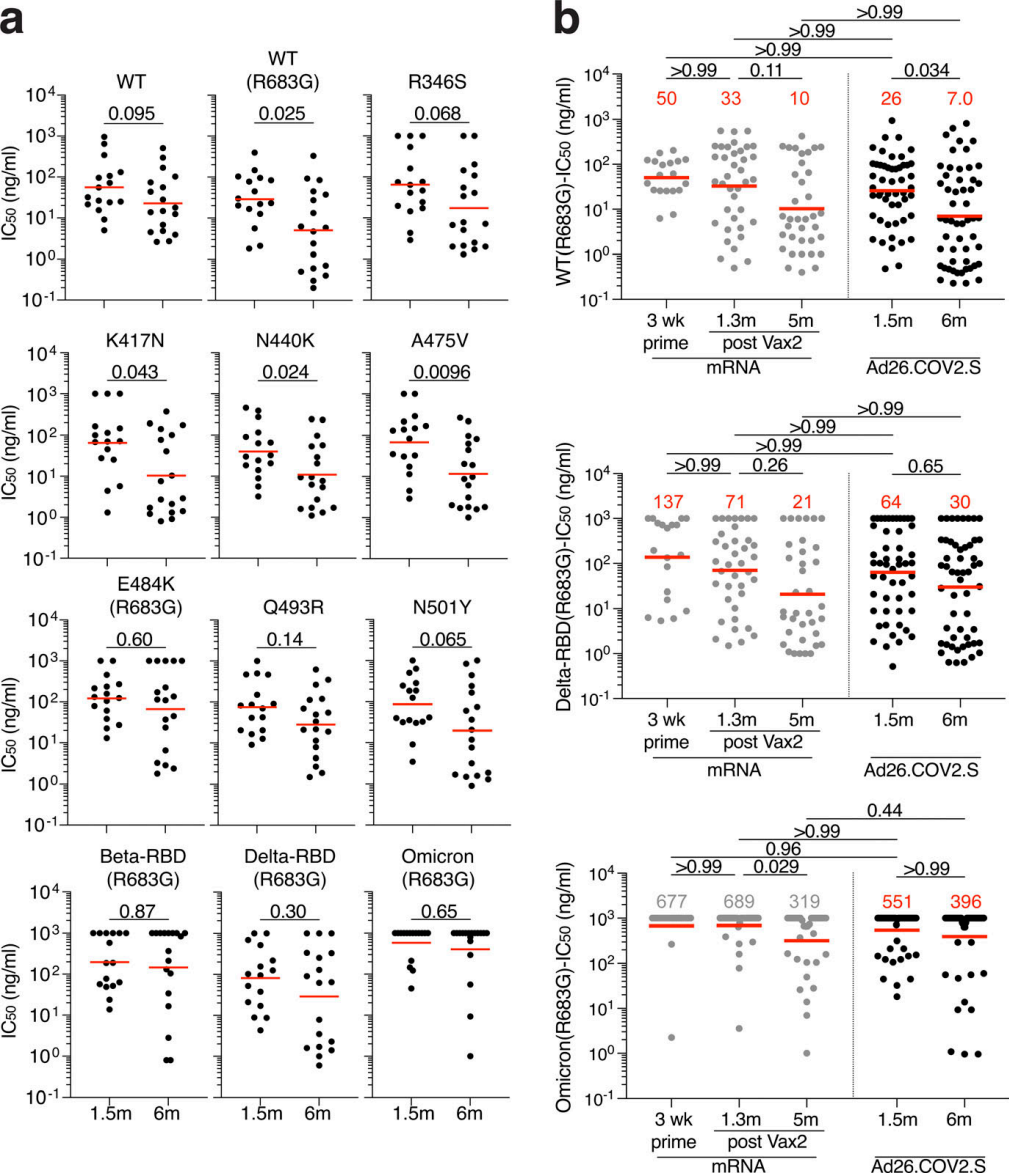
**Breadth. (a)** Graphs showing IC_50_ neutralization activity of antibodies detected at 1.5 mo (*n* = 16) or 6 mo (*n* = 18) against indicated mutant SARS-CoV-2. **(b)** Graphs showing IC_50_ neutralization activity of antibodies at 1.5 mo (*n* = 35) or 6 mo (*n* = 36) against WT (Wuhan-Hu-1 WT), Delta-RBD (L452R/T478K), and Omicron BA.1, compared with mRNA vaccinees at prime and 1.3 and 5 mo after Vax2 (Cho et al., 2021; Muecksch et al., 2022). The E484K, K417N/E484K/N501Y, and L452R/T478K substitutions, as well as the deletions/substitutions corresponding to viral variants, were incorporated into a spike protein that also includes the R683G substitution, which disrupts the furin cleavage site and increases particle infectivity. Neutralizing activity against mutant pseudoviruses was compared with a WT SARS-CoV-2 spike sequence (NC_045512), carrying R683G where appropriate. All experiments were performed at least in duplicate and repeated twice. Red bars and lines indicate geometric mean values. Statistical significance in panel a was determined by two-tailed Mann–Whitney *U* test and in panel b by two-tailed Kruskal–Wallis test with subsequent Dunn’s multiple comparison.

**Figure S4.**
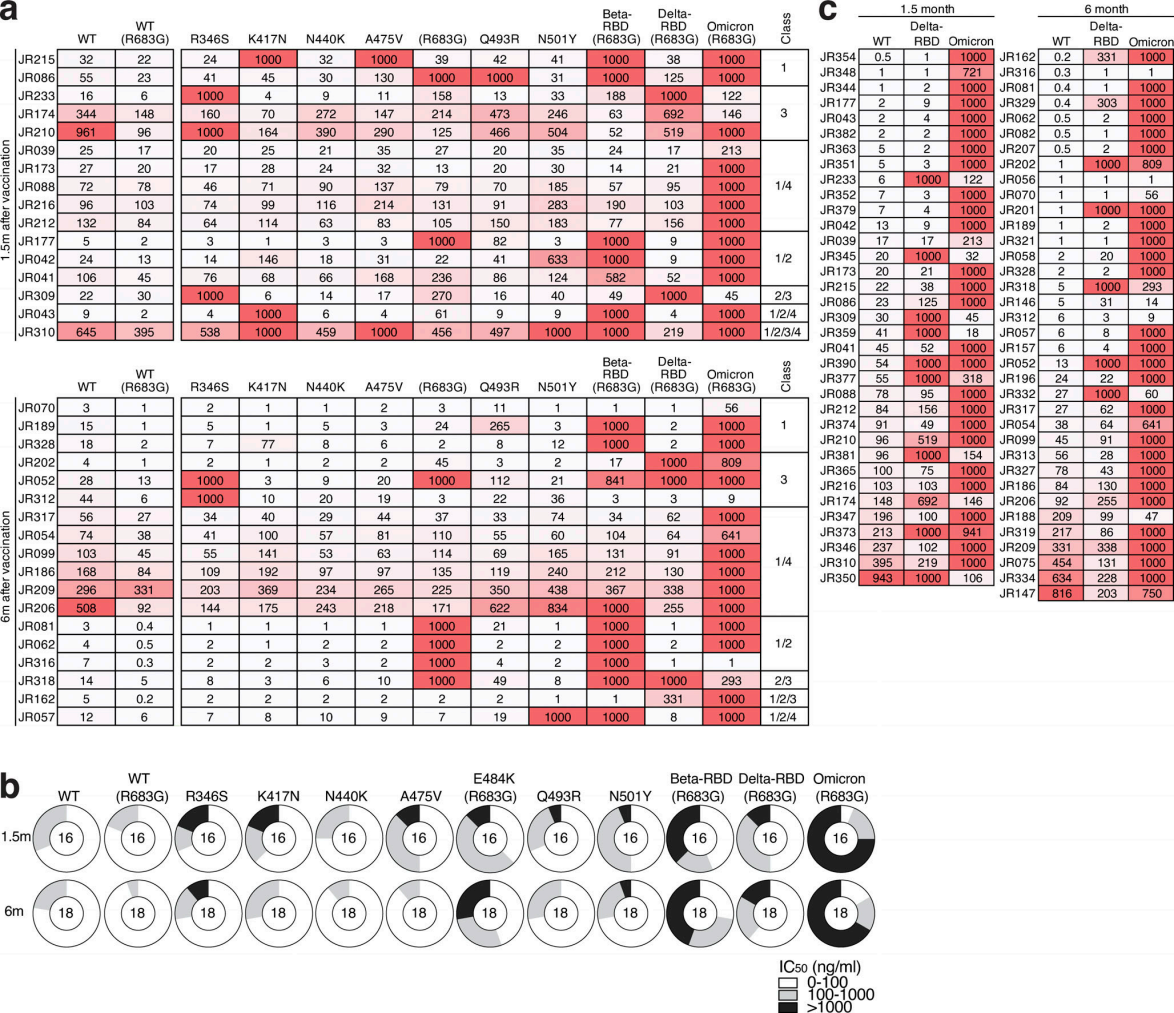
**Neutralizing breadth. (a)** Heatmaps show IC_50_ values of antibodies shown in Fig. 5 a against indicated mutant SARS-CoV-2 pseudoviruses listed across the top. Heatmap ranging from 0.1 to 1,000 ng/ml in white to red. Antibody classes listed to the right were determined by competition BLI (see Fig. 4). **(b)** Ring plots showing fraction of mAbs shown in Fig. 5 a determined to be potently neutralizing (IC_50_ 1–100 ng/ml, white), poorly neutralizing (IC_50_ 100–1,000 ng/ml, gray), or nonneutralizing (IC_50_ > 1,000 ng/ml, black). Mutant or variant SARS-CoV-2 pseudovirus tested is indicated across the top and time point to the left. The number inside the circle indicated the number of antibodies tested. **(c)** Heatmap of antibodies shown in Fig. 5 b, showing IC_50_ values of antibodies detected 1.5 mo (left, *n* = 35) or 6 mo (right, *n* = 36) after vaccination against indicated variant SARS-CoV-2 pseudovirus listed across the top. Heatmap ranging from 0.1 to 1,000 ng/ml in white to red. The E484K, K417N/E484K/N501Y, and L452R/T478K substitutions, as well as the deletions/substitutions corresponding to viral variants, were incorporated into a spike protein that also includes the R683G substitution, which disrupts the furin cleavage site and increases particle infectivity. Neutralizing activities against mutant pseudoviruses were compared with a WT SARS-CoV-2 spike sequence (NC_045512), carrying R683G where appropriate. All experiments were performed at least in duplicate and repeated twice.

We showed that the neutralizing breadth of memory B cell–derived antibodies obtained from convalescent individuals increased significantly after 5 mo (Gaebler et al., 2021; Muecksch et al., 2021; Wang et al., 2021b). However, memory antibodies elicited by mRNA vaccination showed more modest improvement over the same period of time (Cho et al., 2021), which was further increased by a third dose (Muecksch et al., 2022). To determine how the neutralizing breadth of the memory B cell compartment evolves after Ad26.COV2.S vaccination, we analyzed a panel of 34 randomly selected Wuhan-Hu-1–neutralizing antibodies from Ad26.COV2.S vaccinees (*n* = 16 at 1.5 mo and *n* = 18 at 6 mo). Neutralizing activity was measured against SARS-CoV-2 pseudoviruses carrying amino acid substitutions found in variants of concern. Neutralizing breadth improved significantly in Ad26.COV2.S vaccinees against pseudoviruses containing single-amino-acid substitutions found in different SARS-CoV-2 variants (K417N, N440K, and A475V; Fig. 5 a and Fig. S4, a and b). These mutations typically alter the binding and neutralization properties of class 1 and 3 antibodies (Muecksch et al., 2021).

To compare neutralizing responses of memory antibodies against Delta and Omicron BA.1 variants between Ad26.COV2.S and mRNA vaccinees, we tested an additional panel of randomly selected antibodies (*n* = 71) with IC_50_ values <1,000 ng/ml neutralizing activity and tested them against viruses pseudotyped with the variants (Figs. 5 b and S4 c). In contrast to natural infection and mRNA vaccination, there was no improvement in neutralizing activity against Omicron BA.1 1.5–6 mo after Ad26.COV2.S vaccination, whereas responses toward Delta were comparable between the vaccines. Nevertheless, 86% of the 6-mo memory antibodies tested neutralized Delta and 31% neutralized Omicron BA.1 (Fig. S4 c). Thus, 6 mo after vaccination, the memory B cell compartment in Ad26.COV2.S recipients is smaller in size than the RBD-specific memory B cell compartment in mRNA vaccinees (Fig. 2 b) but contains cells with the ability to produce antibodies with comparable activity against Delta and Omicron BA.1.

## Discussion

Administration of a single dose of the Ad26.COV2.S vaccine results in less effective protection against infection than mRNA vaccination and also affords lower levels of protection against severe disease and hospitalization from COVID-19 (Lin et al., 2022; Natarajan et al., 2022; Sadoff et al., 2022). The difference in protective efficacy from infection between the two vaccine modalities has been attributed to significantly lower levels of circulating neutralizing antibodies elicited by the Ad26.COV2.S vaccine (Garcia-Beltran et al., 2022; GeurtsvanKessel et al., 2022). This is in contrast to reports of comparable CD4 and CD8 T cell responses to variants of concern between the two vaccines that persist for ≤8 mo after vaccination (Alter et al., 2021; GeurtsvanKessel et al., 2022; Liu et al., 2022; Tarke et al., 2022; Zhang et al., 2022 *Preprint*). We found that, 5–6 mo after vaccination, there is a 2.5-fold difference in the number of memory B cells produced by the two vaccine modalities. A third mRNA dose further magnifies the difference to nearly sixfold (Muecksch et al., 2022). Nevertheless, the antibodies encoded by the individual memory B cells show similar levels of activity against Wuhan-Hu-1, Delta, and Omicron BA.1. The ability of these cells to respond rapidly to viral challenge may account in part for the partial protection against severe disease by Ad26.COV2.S vaccination.

Circulating antibodies are produced from plasma cells that are selected in germinal centers and extrafollicular foci from a diverse cohort of follicular B cells based primarily on their affinity for antigen (Phan et al., 2006; Weisel et al., 2016). Many of the plasma cells produced during the early stages of the immune response are short-lived, resulting in a transient early peak in circulating antibody levels (Wrammert et al., 2008). Memory B cells develop in the same two microanatomic compartments, but their development is regulated by an entirely different cellular and molecular program (Choi and Crotty, 2021; Inoue et al., 2021; Laidlaw and Cyster, 2021; Papa and Vinuesa, 2018; Victora and Nussenzweig, 2022). As a result, memory B cells are long-lived and express a diverse collection of antibodies with differing affinities, neutralizing activities, and breadths (Viant et al., 2020; Victora and Nussenzweig, 2022).

The relatively poor plasma binding and neutralizing titers elicited by the Ad26.COV2.S vaccine compared with mRNA vaccines points to more modest elicitation of plasma cell responses by Ad26.COV2.S. In addition, the number of RBD-specific memory B cells elicited by the single-dose Ad26.COV2.S vaccine is smaller than that elicited by two doses of the mRNA vaccines at all time points examined. A third mRNA booster vaccination amplifies this difference. Similar to mRNA vaccinees (Amanat et al., 2021), NTD-specific memory B cells elicited by Ad26.COV2.S vaccination show little neutralizing activity but have the potential to contribute to protection through Fc-mediated effector pathways (Beaudoin-Bussieres et al., 2022). In contrast, the neutralizing potency and breadth of RBD-specific memory cells develop rapidly after Ad26.COV2.S vaccination, and the memory antibodies elicited by the two vaccine modalities display comparable potency and breadth against Wuhan-Hu-1 and Delta at both 1.5 and 6 mo after vaccination. Activity against Omicron BA.1 was lower after Ad26.COV2.S, but the difference was not statistically significant. Booster vaccinations are essential for eliciting higher neutralizing titers against Omicron BA.1 and BA.2 and better protection against Omicron BA.1 (Accorsi et al., 2022; Bowen et al., 2022 *Preprint*; Gray et al., 2022). This is important to consider, especially with the rapid evolution of new variants of concern (Tegally et al., 2022 *Preprint*) and continuing efforts to provide booster vaccinations to Ad26.COV2.S vaccinees, of whom only 20% received a heterologous mRNA boost (Centers for Disease Control and Prevention, 2022).

Class 1 and 2 antibodies develop early after infection or mRNA immunization and are generally more potent than classes 3 and 4, because they interfere directly with the interaction between the SARS-CoV-2 RBD and its cellular receptor ACE2 (Barnes et al., 2020a; Muecksch et al., 2021; Wang et al., 2021b). However, this renders class 1 and 2 antibodies highly sensitive to amino acid substitutions within the ACE2 binding ridge of the RBD found in many SARS-CoV-2 variants (Muecksch et al., 2021; Wang et al., 2021b). The epitopes targeted by classes 3 and 4 are generally more conserved, and antibodies binding to these epitopes may be more broadly reactive. Class 3 and 4 antibodies develop earlier in Ad26.COV2.S than in mRNA vaccinees, leading to a more diverse early B cell memory response. Nevertheless, continued evolution is a feature of memory B cell responses to both vaccine modalities, and they become comparable in this respect after 5–6 mo.

Neutralizing antibodies are the best correlate of protection, and when provided early, they are also therapeutic against COVID-19 (Gupta et al., 2021; Li et al., 2022; O’Brien et al., 2021; Taylor et al., 2021; Weinreich et al., 2021). Although memory B cells are quiescent and do not contribute to the pool of circulating antibodies under steady-state conditions, they can be recalled rapidly upon challenge to develop into antibody-producing cells (Amanna et al., 2007; Mesin et al., 2020). Our observations show that a diverse memory B cell compartment develops in response to the Ad26.COV2.S vaccine, including a subset of cells that express antibodies that potently neutralize antigenically divergent variants, including Delta and Omicron BA.1.

Rapid activation of these cells and antibody production upon SARS-CoV-2 infection may explain why the Ad26.COV2.S vaccine is partially effective at providing protection against severe disease after breakthrough infection, and priming with this vaccine supports robust responses after heterologous boosting with mRNA vaccines (Atmar et al., 2022; GeurtsvanKessel et al., 2022; Natarajan et al., 2022).

## Materials and methods

### Study participants

Participants were healthy volunteers who had previously received one dose of the Janssen (Ad26.COV2.S) vaccine against WT (Wuhan-Hu-1) strain of SARS-CoV-2. For this study, participants were recruited for serial blood donations at the Rockefeller University Hospital in New York between April 26, 2021 and August 16, 2021. Eligible participants (*n* = 18) were healthy adults with no history of infection with SARS-CoV-2 during or before the observation period (as determined by clinical history and confirmed through serology testing) who had received only one dose of the SARS-CoV-2 Janssen Ad26.COV2.S vaccine. Exclusion criteria include presence of clinical signs and symptoms suggestive of acute infection, a positive RT-PCR result for SARS-CoV-2 in saliva, or positive COVID-19 serology. Participants presented to the Rockefeller University Hospital for blood sample collection and were asked to provide details of their vaccination regimen, possible side effects, comorbidities, and possible COVID-19 history. Clinical data collection and management were carried out using the software iRIS by iMedRIS (v11.02). All participants provided written informed consent before participation in the study. The study was conducted in accordance with Good Clinical Practices and all relevant ethics regulations, and the protocol (DRO-1006) for studies with human participants was approved by the Institutional Review Board of the Rockefeller University. For detailed participant characteristics, see Table S1. All data was compared with demographically matched, previously published cohorts of mRNA vaccinees or convalescent individuals (Table S2; Cho et al., 2021; Gaebler et al., 2021; Muecksch et al., 2022; Robbiani et al., 2020; Wang et al., 2021c).

### Blood sample processing and storage

Peripheral blood mononuclear cells obtained from samples collected at Rockefeller University were purified as previously reported by gradient centrifugation and stored in liquid nitrogen in the presence of FCS and DMSO (Gaebler et al., 2021; Robbiani et al., 2020). Heparinized plasma and serum samples were aliquoted and stored at −20°C or less. Before experiments, aliquots of plasma samples were heat-inactivated (56°C for 30 min) and then stored at 4°C.

### ELISAs

ELISAs (Amanat et al., 2020; Grifoni et al., 2020) to evaluate antibodies binding to SARS-CoV-2 RBD or NTD were performed by coating of high-binding 96-half-well plates (Corning 3690) with 50 μl per well of a 1 μg/ml protein solution in PBS overnight at 4°C. Plates were washed six times with washing buffer (1× PBS with 0.05% Tween-20; Sigma-Aldrich) and incubated with 170 μl per well blocking buffer (1× PBS with 2% BSA and 0.05% Tween-20; Sigma-Aldrich) for 1 h at room temperature. Immediately after blocking, mAbs or plasma samples were added in PBS and incubated for 1 h at room temperature. Plasma samples were assayed at a 1:66 starting dilution and 10 additional threefold serial dilutions. Monoclonal antibodies were tested at 10 μg/ml starting concentration and 10 additional fourfold serial dilutions. Plates were washed six times with washing buffer and then incubated with anti-human IgG, IgM, or IgA secondary antibody conjugated to HRP (Jackson ImmunoResearch 109-036-088 and 109-035-129 and Sigma-Aldrich A0295) in blocking buffer at a 1:5,000 (IgM and IgG) or 1:3,000 (IgA) dilution. Plates were developed by addition of the HRP substrate, 3,3′,5,5′-tetramethylbenzidine (Thermo Fisher Scientific) for 10 min (plasma samples) or 4 min (mAbs). The developing reaction was stopped by adding 50 μl of 1 M H_2_SO_4_, and absorbance was measured at 450 nm with an ELISA microplate reader (FluoStar Omega; BMG Labtech) and analyzed with Omega and Omega MARS software. For plasma samples, a positive control (plasma from participant COV72, diluted 66.6-fold and 10 additional threefold serial dilutions in PBS) was added to every assay plate for normalization. The average of its signal was used for normalization of all the other values on the same plate with Excel software before calculating the area under the curve using Prism v9.1 (GraphPad). Negative controls of prepandemic plasma samples from healthy donors were used for validation (for more details, see Robbiani et al., 2020). For mAbs, the ELISA EC_50_ was determined using four-parameter nonlinear regression (GraphPad Prism v9.1). EC_50_ values >1,000 ng/ml for RBD binding were considered binders; EC_50_ values >10,000 ng/ml for NTD binding were considered nonbinders.

### Proteins

The mammalian expression vector encoding the RBD of SARS-CoV-2 (GenBank MN985325.1; S-protein residues 319–539) was previously described (Barnes et al., 2020b). Mammalian expression vector encoding the SARS-CoV-2 Wuhan-Hu-1 NTD (GenBank MN985325.1; S-protein residues 14–307) was previously described (Wang et al., 2022 *Preprint*).

### SARS-CoV-2 pseudotyped reporter virus

A panel of plasmids expressing RBD-mutant SARS-CoV-2 spike proteins in the context of pSARS-CoV-2-S_Δ19_ has been described (Cho et al., 2021; Muecksch et al., 2021; Wang et al., 2021c; Weisblum et al., 2020). Variant pseudoviruses resembling SARS-CoV-2 variants Beta (B.1.351), B.1.526, Delta (B.1.617.2), and Omicron BA.1 (B.1.1.529) have been described (Cho et al., 2021; Schmidt et al., 2022; Wang et al., 2021b) and were generated by introduction of substitutions using synthetic gene fragments (IDT) or overlap extension PCR-mediated mutagenesis and Gibson assembly. Specifically, the variant-specific deletions and substitutions introduced were Beta: D80A, D215G, L242H, R246I, K417N, E484K, N501Y, D614G, A701V; Delta: T19R, Δ156–158, L452R, T478K, D614G, P681R, D950N; and Omicron BA.1: A67V, Δ69–70, T95I, G142D, Δ143–145, Δ211, L212I, ins214EPE, G339D, S371L, S373P, S375F, K417N, N440K, G446S, S477N, T478K, E484A, Q493K, G496S, Q498R, N501Y, Y505H, T547K, D614G, H655Y, H679K, P681H, N764K, D796Y, N856K, Q954H, N969H, N969K, L981F.

The E484K, K417N/E484K/N501Y, and L452R/T478K substitutions, as well as the deletions/substitutions corresponding to variants of concern listed above, were incorporated into a spike protein that also includes the R683G substitution, which disrupts the furin cleavage site and increases particle infectivity. Neutralizing activity against mutant pseudoviruses was compared with a WT SARS-CoV-2 spike sequence (NC_045512), carrying R683G where appropriate.

SARS-CoV-2 pseudotyped particles were generated as previously described (Robbiani et al., 2020; Schmidt et al., 2020). Briefly, 293T (CRL-11268) cells were obtained from ATCC, and the cells were transfected with pNL4-3 ΔEnv-nanoluc and pSARS-CoV-2-S_Δ19_. Particles were harvested 48 h after transfection, filtered, and stored at −80°C.

### Pseudotyped virus neutralization assay

Four- to fivefold serially diluted prepandemic negative control plasma samples from healthy donors, plasma from individuals who received Ad26.COV2.S vaccines, or mAbs were incubated with SARS-CoV-2 pseudotyped virus for 1 h at 37°C. The mixture was subsequently incubated with 293T_Ace2_ cells (Robbiani et al., 2020; for all WT neutralization assays) or HT1080Ace2 cl14 cells (for all mutant panels and variant neutralization assays; Wang et al., 2021c) for 48 h, after which cells were washed with PBS and lysed with Luciferase Cell Culture Lysis 5× reagent (Promega). Nanoluc Luciferase activity in lysates was measured using the Nano-Glo Luciferase Assay System (Promega) with the Glomax Navigator (Promega) or ClarioStar multimode microplate reader (BMG). The relative luminescence units were normalized to those derived from cells infected with SARS-CoV-2 pseudotyped virus in the absence of plasma or mAbs. The NT_50_, IC_50_, and 90% inhibitory concentration (IC_90_) for mAbs were determined using four-parameter nonlinear regression (least-squares regression method without weighting; constraints: top = 1, bottom = 0; GraphPad Prism).

### Biotinylation of viral protein for use in flow cytometry

Purified and Avi-tagged SARS-CoV-2 Wuhan-Hu-1 RBD and NTD were biotinylated using the Biotin-Protein Ligase-BIRA kit according to the manufacturer’s instructions (Avidity) as described before (Robbiani et al., 2020). Ovalbumin (A5503-1G; Sigma-Aldrich) was biotinylated using the EZ-Link Sulfo-NHS-LC-Biotinylation kit according to the manufacturer’s instructions (Thermo Fisher Scientific). Biotinylated ovalbumin was conjugated to streptavidin-BB515 (564453; BD). RBD was conjugated to streptavidin-PE (554061; BD Biosciences) and streptavidin-AF647 (405237; BioLegend; Robbiani et al., 2020). NTD was conjugated to streptavidin-BV421 (405225; Biolegend) and streptavidin-BV711 (563262; BD Biosciences).

### Flow cytometry and single-cell sorting

Single-cell sorting by flow cytometry was described previously (Robbiani et al., 2020). Briefly, peripheral blood mononuclear cells were enriched for B cells by negative selection using a pan-B-cell isolation kit according to the manufacturer’s instructions (130-101-638; Miltenyi Biotec). The enriched B cells were incubated in FACS buffer (1× PBS, 2% FCS, and 1 mM EDTA) with the following anti-human antibodies (all at 1:200 dilution): anti-CD20-PECy7 (335793; BD Biosciences), anti-CD3-APC-eFluro780 (47-0037-41; Invitrogen), anti-CD8-APC-eFluor780 (47-0086-42; Invitrogen), anti-CD16-APC-eFluor780 (47-0168-41; Invitrogen), and anti-CD14-APC-eFluor780 (47-0149-42; Invitrogen); Zombie NIR (423105; BioLegend); and fluorophore-labeled Wuhan-Hu-1 RBD, NTD, and ovalbumin (Ova) for 30 min on ice. AccuCheck Counting Beads (PCB100; Life Technologies) were added to each sample according to the manufacturer’s instructions. Single CD3^−^CD8^−^CD14^−^CD16^−^CD20^+^Ova^−^ B cells that were either RBD-PE^+^RBD-AF647^+^ or NTD-BV711^+^NTD-BV421^+^ were sorted into individual wells of 96-well plates containing 4 μl of lysis buffer (0.5× PBS, 10 mM dithiothreitol, and 3,000 units/ml RNasin ribonuclease inhibitors [N2615; Promega]) per well using a FACS Aria III and FACSDiva software (Becton Dickinson) for acquisition and FlowJo for analysis. The sorted cells were frozen on dry ice and then stored at −80°C or immediately used for subsequent RNA reverse transcription. For B cell phenotype analysis, in addition to the above antibodies, B cells were also stained with the following anti-human antibodies (all at 1:200 dilution): anti-IgD-BV650 (740594; BD), anti-CD27-BV786 (563327; BD Biosciences), anti-CD19-BV605 (302244; BioLegend), anti-CD71^−^ PerCP-Cy5.5 (334114; BioLegend), anti-IgG-PECF594 (562538; BD), anti-IgM-AF700 (314538; BioLegend), and anti-IgA-Viogreen (130-113-481; Miltenyi Biotec).

### Antibody sequencing, cloning, and expression

Antibodies were identified and sequenced as described previously (Robbiani et al., 2020; Wang et al., 2021a). In brief, RNA from single cells was reverse transcribed (SuperScript III Reverse Transcriptase, 18080-044; Invitrogen), and the cDNA was stored at −20°C or used for subsequent amplification of the variable IGH, IGL, and IGK genes by nested PCR and Sanger sequencing. Sequence analysis was performed using MacVector. Amplicons from the first PCR reaction were used as templates for sequence- and ligation-independent cloning into antibody expression vectors. Recombinant mAbs were produced and purified as previously described (Robbiani et al., 2020).

### BLI

BLI assays were performed as previously described (Robbiani et al., 2020). Briefly, we used the Octet Red instrument (ForteBio) at 30°C with shaking at 1,000 rpm. Epitope binding assays were performed with protein A biosensor (18-5010; ForteBio), following the manufacturer’s protocol “classical sandwich assay” as follows: (1) sensor check: sensors immersed for 30 s in buffer alone (18-1105; buffer ForteBio); (2) capture first antibody: sensors immersed for 10 min with Ab1 at 10 µg/ml; (3) baseline: sensors immersed for 30 s in buffer alone; (4) blocking: sensors immersed for 5 min with IgG isotype control at 10 µg/ml; (5) baseline: sensors immersed for 30 s in buffer alone; (6) antigen association: sensors immersed for 5 min with RBD at 10 µg/ml; (7) baseline: sensors immersed for 30 s in buffer alone; and (8) association Ab2: sensors immersed for 5 min with Ab2 at 10 µg/ml. Curve fitting was performed using Octet Data analysis software (ForteBio).

### Computational analyses of antibody sequences

Antibody sequences were trimmed based on quality and annotated using Igblastn v1.14 with IMGT domain delineation system. Annotation was performed systematically using Change-O toolkit v0.4.540 (Gupta et al., 2015). Clonality of heavy and light chain was determined using DefineClones.py implemented by Change-O v0.4.5 (Gupta et al., 2015). The script calculates the Hamming distance between each sequence in the data set and its nearest neighbor. Distances are subsequently normalized and to account for differences in junction sequence length, and clonality is determined based on a cutoff threshold of 0.15. Heavy and light chains derived from the same cell were subsequently paired, and clonotypes were assigned based on their V and J genes using in-house R and Perl scripts. All scripts and the data used to process antibody sequences are publicly available on GitHub (https://github.com/stratust/igpipeline/tree/igpipeline2_timepoint_v2).

The frequency distributions of human V genes in anti-SARS-CoV-2 antibodies from this study was compared with 131,284,220 IgH and IgL sequences generated by Soto et al. (2019) and downloaded from cAb-Rep (Guo et al., 2019), a database of human shared BCR clonotypes available at https://cab-rep.c2b2.columbia.edu/. Based on the 150 distinct V genes that make up the 1,099 analyzed sequences from Ig repertoire of the six participants in this study, we selected the IgH and IgL sequences from the database that were partially coded by the same V genes and counted them according to the constant region. The frequencies shown in Fig. S3 are relative to the source and isotype analyzed. We used the two-sided binomial test to check whether the number of sequences belonging to a specific *IGHV* or *IGLV* gene in the repertoire was different according to the frequency of the same IgV gene in the database. Adjusted P values were calculated using the false discovery rate correction. Significant differences are denoted with asterisks.

Nucleotide somatic hypermutation (SHM) and complementarity-determining region 3 (CDR3) length were determined using in-house R and Perl scripts. For SHMs, *IGHV* and *IGLV* nucleotide sequences were aligned against their closest germlines using Igblastn, and the number of differences was considered the nucleotide mutations. The average number of mutations for V genes was calculated by dividing the sum of all nucleotide mutations across all participants by the number of sequences used for the analysis.

### Data presentation

Figures were arranged in Adobe Illustrator 2022.

### Online supplemental material

Fig. S1 shows plasma IgM and IgA RBD-binding activity after vaccination. Fig. S2 shows flow cytometry gating strategy to phenotype or sort RBD- and NTD-binding memory B cells after vaccination. Fig. S3 shows frequency of V gene usage of RBD- and NTD-binding memory B cells after vaccination. Fig. S4 shows additional information on neutralizing breadth of antibodies cloned from RBD-specific memory B cells. Table S1 details individual characteristics for participants who received Ad26.COV2.S. Table S2 provides a cohort summary of all vaccinated individuals. Table S3 details plasma neutralization activity against variant SARS-CoV-2. Table S4 details sequence information of all characterized RBD- and NTD-binding memory B cells from Ad26.COV2.S vaccinated individuals. Table S5 provides information of all recombinant mAbs cloned from RBD-binding B cells. Table S6 provides information of all recombinant mAbs cloned from NTD-binding B cells. Table S7 provides epitope specificity of mAbs.

## Supplementary Material

Table S1 lists individual participant characteristics.

Table S2 shows the cohort summary.

Table S3 lists the neutralization activity of plasma against variant SARS-CoV-2 pseudoviruses.

Table S4 lists sequences of anti–SARS-CoV-2 RBD- and NTD-specific antibodies.

Table S5 lists sequences, RBD binding, and neutralization of cloned recombinant antibodies.

Table S6 lists sequences, NTD binding, and neutralization of cloned recombinant antibodies.

Table S7 lists epitope specificity of mAbs.

## Data Availability

Data are provided in Tables S1, S2, S3, S4, S5, S6, and S7. The raw sequencing data and computer scripts associated with Fig. 2 have been deposited at Github (https://github.com/stratust/igpipeline/tree/igpipeline2_timepoint_v2). This study also uses data from DeWitt et al. (2016), PDB (6VYB and 6NB6), cAb-Rep (https://cab-rep.c2b2.columbia.edu/), Sequence Read Archive (accession no. SRP010970), and Soto et al. (2019). Computer code to process the antibody sequences is available at GitHub (https://github.com/stratust/igpipeline/tree/igpipeline2_timepoint_v2).
